# ﻿New morphological and biological contributions to adults and immature forms of *Pissonotusparaguayensis* (Fulgoromorpha, Delphacidae) in wetlands of Argentina

**DOI:** 10.3897/zookeys.1188.113350

**Published:** 2024-01-08

**Authors:** Ana M. Marino de Remes Lenicov, Ana C. Faltlhauser, Alvaro Foieri, Nicolas A. Salinas, M. Cristina Hernández, Alejandro J. Sosa

**Affiliations:** 1 División Entomología, Facultad de Ciencias Naturales y Museo, Universidad Nacional de La Plata, La Plata, Argentina Consejo Nacional de Investigaciones Científicas y Técnicas Buenos Aires Argentina; 2 Consejo Nacional de Investigaciones Científicas y Técnicas, Ciudad Autónoma de Buenos Aires, Argentina Universidad Nacional de La Plata La Plata Argentina; 3 Fundación para el Estudio de Especies Invasivas, Hurlingham, Buenos Aires, Argentina Fundación para el Estudio de Especies Invasivas Buenos Aires Argentina; 4 Universidad de Buenos Aires, Facultad de Ciencias Exactas y Naturales, Departamento de Ecología, Genética y Evolución, CABA, Buenos Aires, Argentina Universidad de Buenos Aires Buenos Aires Argentina; 5 Comisión de Investigaciones Científicas, La Plata, Buenos Aires, Argentina Comisión de Investigaciones Científicas La Plata Argentina

**Keywords:** Biology, female wing polymorphism, host range, immature stages key, *Ludwigia*, morphology, new record distribution, planthopper

## Abstract

In the search for insects as biological control agents for the water primrose, the delphacid *Pissonotusparaguayensis* (Delphacidae) was found on Ludwigiagrandiflorasubsp.hexapetala (Onagraceae) in a wetland of Central East Argentina. The morphology of the unknown females (brachypterous and macropterous) and immature stages are described and illustrated. Adults and nymphs were collected in wetlands of Del Plata River Basin, from Buenos Aires to the northeastern part of Argentina. A rearing methodology was developed to perform biological studies. Both winged forms and structural features of the female genitalia are described for the first time at the genus level. Eggs and immature stages are described and keyed; fifth nymphal instars may be easily recognised by the yellowish colouration, blackish on dorsal of head, thorax and abdomen with conspicuous yellowish pits, ventrally only darkened on base of frons extended to lower level of eyes and dorsal surface of antennomeres I and II, and legs with distinctive black marks at femoro-tibial joint and apex. The geographical distribution is updated, expanding its range into Argentina, making Buenos Aires the southernmost limit of the genus in America. Biological information of the species is also reported here: life cycle, fecundity, oviposition behaviour, and host plant. Field observations showed that *P.paraguayensis* breeds, feeds, and causes damage to L.g.subsp.hexapetala. This delphacid presents a certain degree of specificity to the *Ludwigia* species in the Jussiaea section in host specificity tests. More studies are required to test this species as a potential biological control agent.

## ﻿Introduction

The water primrose, *Ludwigiag*. subsp. hexapetala (Hook. & Arn.) G.L. Nesom & Kartesz, Michx. (Onagraceae) is a native South American plant (Wagner et al. 2007) that has been introduced in many countries around the world, invading both aquatic and riparian ecosystems, and is considered among the most aggressive weeds in the world (Thouvenot et al. 2013). In the search for insects as biological control agents for water primrose, a delphacid *Pissonotusparaguayensis* Bartlett, 2000 was found on this plant in a wetland of Central East Argentina. This delphacid species has a detailed description of the brachypter male but little is known about the female form and nothing about its immature stages and biology.

*Pissonotus* Van Duzee, 1897 is a genus widespread in North America, from southern Canada, with a few species in the Caribbean and Neotropics (Bartlett and Deitz 2000). Since the description of the first eight North American species by Van Duzee, several authors have studied this genus, describing new species or relocating them within *Pissonotus* from other genera (Spooner 1912; Crawford 1914; Metcalf 1923; Muir and Giffard 1924; Oman 1947). The first revision of the genus was carried out by Morgan and Beamer (1949) and more recently Bartlett and Deitz (2000) presented an integrative contribution about aspects of its taxonomy, phylogenetic relationships, distribution and host plant associations with descriptions and keys for all 43 recognised species. This revision shows that colour and the morphological features provided mostly by male genitalia, particularly the shape of the paired median processes of the pygofer, are distinctive among the species. Although wing polymorphism is frequent in delphacid populations, brachypterism in both sexes prevails in more than half of the species of the genus, and the macropters are more erratically found (Bartlett and Deitz 2000). The wing dimorphism, as a life history strategy in *Pissonotus*, was investigated by Denno et al. (1991) and Denno and Roderick (1990), who used this feature to test hypotheses concerning the effects of persistence and architectural complexity of habitat on the occurrence and dispersion of populations.

Further studies are needed on immature stages of the genus, their morphology, and behaviour. Of North American species, only *P.delicatus* Van Duzee is known from immature stages (Wilson and Tsai 1991). Knowledge of immature morphology also adds new characters for identification, taxonomic diagnosis, and phylogenetic analysis as proposed by Yang and Fang (1993), Chen and Yang (1995), and Emeljanov (1996, 2002). This information can help estimate their richness, diversity and particularly, their association with invasive host plants.

Bartlett and Deitz (2000) reported aspects of the geographical distribution pattern and some information about host plants of *Pissonotus* spp. In same contribution, mentioned that 91% of the total species occur in North America while 6% (3 species) are restricted to South American environments, two of them, *P.boliviensis* Bartlett and *P.neotropicus* (Muir) are recorded from Argentina.

Plant associations are relatively rare, and only 20 of 43 species have their hosts recorded, although they have been confirmed in few species. Those records suggest that *Pissonotus* feeds primarily on dicots, especially Asteraceae with some few exceptions on other aquatic host plants outside of this family. For example, *P.boliviensis*, distributed in Central South America, was reported in Bolivia on Pontederiaceae: *Pontederiarotundifolia* L. and *Pontederia* sp. as hosts; and *P.piceus* (Van Duzze), widely distributed in Central and Eastern United States of America (USA), Central America, Northern South America, and the Caribbean were recorded in the USA on Onagraceae: *Ludwigiapeploides* (H.B.K.) Raven (as *Jussiaeadiffusa* Forskal) (Morgan and Beamer 1949), *L.grandiflora* (Michx.) Greuter & Burdet and *L.uruguayensis* (Camb.) Hara (now L.g.subsp.grandiflora (Michx.) Greuter & Burdet and L.g.subsp.hexapetala). Additionally, this last species has several other hosts, including species in the Poaceae and non-Poaceae families (Wilson et al. 1994; Bartlett and Deitz 2000). Particularly in Argentina, eight species of Delphacidae have been the subjects of research, specifically investigating their level of association with macrophytes (Sosa et al. 2004, 2007; Mariani et al. 2013; Remes Lenicov and Cabrera Walsh 2013). Among these species, information regarding their life cycles has been gathered for three of them (Sosa et al. 2005; Mariani et al. 2007; Marino de Remes Lenicov et al. 2017).

During an ongoing search for native South American herbivores as potential biological control agents against L.g.subsp.hexapetala, three unidentified delphacids were collected (Hernández and Cabrera Walsh 2014). Those specimens were examined and are reported here as *P.paraguayensis*.

Herein this study presents new morphological features of the female and immature stages of *P.paraguayensis* contributing to the complete description of the species including a standard COI barcode sequence and a key to the instars. It also documents new distribution records, rearing methodology and biological information based on field and laboratory observations. Since this delphacid could be considered a biological control candidate, the host range is estimated.

## ﻿Materials and methods

### ﻿Insect collection

Between 2009 and 2011, specimens of delphacids (adults and nymphs) were collected by Hernández and Cabrera Walsh (2014) feeding on *L.g*. subsp.hexapetala. Samples were identified as *P.paraguayensis*, collection field trips were resumed in 2019 in areas where L.g.subsp.hexapetala is most commonly distributed (from Buenos Aires to the northeastern part of Argentina). Adults and nymphs of *P.paraguayensis* were collected both, by aspirating individuals from the leaves and stems of field host plants, and laboratory colonies. Specimens were preserved in 70% EtOH for morphological studies. Live adults and immature specimens from the lower Delta were used to describe colour patterns (Table 1).

**Table 1.** First distribution sites for *Pissonotusparaguayensis* in Argentina (geographical coordinates in Degrees, Minutes, and Seconds (DMS)).

Location	Province	GPS coordinates	Observation date	Status	Host plant
Arroyo Ceibal	Santa Fe	28°42'57.7"S, 59°26'21.7"W		Hernández et al. 2014*	Ludwigiagrandiflorasubsp.hexapetala
Médanos	Entre Ríos	33°25'25.4"S, 59°5'47.4"W	30–03–2022	new	Ludwigiagrandiflorasubsp.hexapetala
Islas del Ibicuy	Entre Ríos	33°41'50.4"S, 58°55'12.3"W	05–02–2022	new	Ludwigiagrandiflorasubsp.hexapetala
Brazo Largo	Entre Ríos	33°51'53.4"S, 58°52'59.4"W	05–03–2022	new	Ludwigiagrandiflorasubsp.hexapetala
San Pedro	Buenos Aires	34°40'46.9"S, 59°38'52.4"W	06–02–2022	new	Ludwigiagrandiflorasubsp.hexapetala
Otamendi	Buenos Aires	34°03'48.2"S, 58°49'19.6"W		Hernández et al. 2014*	Ludwigiagrandiflorasubsp.hexapetala
Luján	Buenos Aires	34°33'57.6"S, 59°4'4.8"W	11–03–2022	new	Ludwigiagrandiflorasubsp.hexapetala
Dique Luján	Buenos Aires	34°21'17.1"S, 58°41'13.0"W	20–07–2023	new	Ludwigiagrandiflorasubsp.hexapetala
Magdalena	Buenos Aires	35°3'48.9"S, 57°33'14.9"W	03–11–2021	new	Ludwigiagrandiflorasubsp.hexapetala
La Plata	Buenos Aires	35°0'50.2"S, 58°0'36.6"W	03–01–2023	new	Ludwigiagrandiflorasubsp.hexapetala

* reported as sp. 1, sp. 2, and sp. 3.

### ﻿Rearing methodology

**Figure 1.**
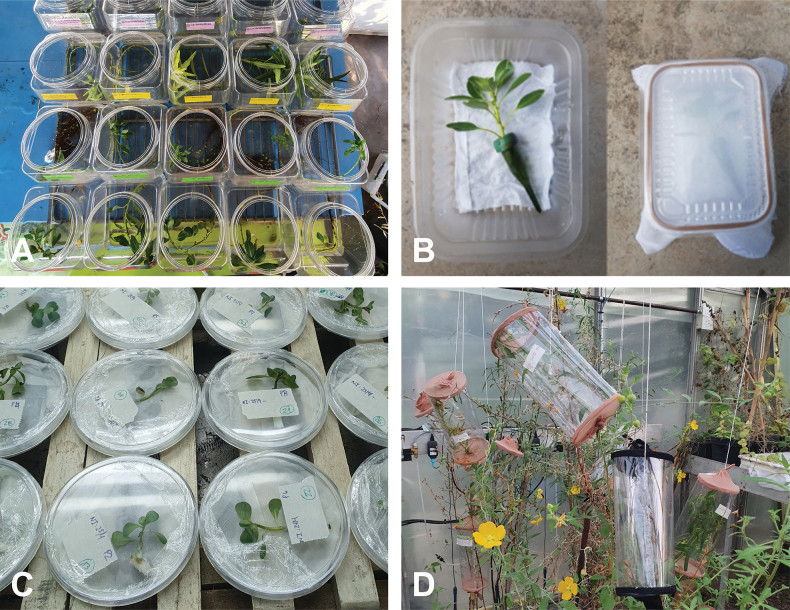
Different rearing and test devices for *Pissonotusparaguayensis***A** device for aquatic plants utilised for rearing and host range tests **B** device for rearing and tests with plants in water-pics **C** small petri dishes with *L.g*. susp. *hexapetala* utilised in life cycle study **D** hanging plastic cylinders utilised in host range test for terrestrial plants.

During summer months (December to March), general culture consisted of outdoor pools (1×1.5 m) with L.g.subsp.hexapetala plants brought from the field and enclosed in square meshed cages. In cooler months (May to August) or if there is no availability of greenhouse conditions, *P.paraguayensis* can also be reared in small containers inside rearing chambers at FuEDEI facilities. We were able to maintain colonies in two types of containers with different water availability and size. One to three L cylindrical plastic containers with polyester gauze or nylon covers and enough water to irrigate the plant roots (Fig. 1A). Here, 10–20 cm plants can be placed vertically but insects are harder to observe or retrieve. The second device consisted of 20×15×5 cm rectangular plastic containers with pierced plastic lids and polyester gauze. Stems were placed horizontally and inside water picks for irrigation (Fig. 1B). Excess condensation on the walls is not recommended.

### ﻿Morphology

Identification of *P.paraguayensis* was based on the original description and photographs of the holotype from the Arizona State University, Lois B. O’Brien Collection (**ASU: ASULOB**). Female (brachypters and macropters) specimens, captured in the same place and host plant were structurally matched with available males, then used for dissections, images and complementary descriptions are deposited in the Museo de La Plata entomological collection (**MLP**). Immature stages: description was based on 24-hour-old nymphs from laboratory colonies. Specimens were submitted to 95% ethyl ether to preserve colour, cleared in cold 10% KOH solution and fixed in Faure liquid for microscopic examination and illustration. Eggs: obtained by dissecting oviposition scars. First instar: described in detail, focused on major differences are highlighted for older stages and also between presumptive brachypterous and macropterous forms of the fifth instar. Measurements: range and median, given in mm, taken from anaesthetised specimens. Dimensions were expressed as **L.**: total body length (from the tip of vertex to the tip of abdomen), **W.**: maximum body width (across the widest part of the mesothorax), t.l.: thoracic length (from the anterior margin of the pronotum to the posterior margin of the metanotum along the midline). Other measurements are relative. Morphological terminology and measurements of adults follow Bartlett and Deitz (2000), but the wing venation and female genitalia refer to Bourgoin et al. (2015) and Asche (1985) respectively. Carination and arrangement of pits of nymphs generally follows Vilbaste (1968) updated after by Yang and Yeh (1994). External morphology was observed with a Leica EZ5 stereomicroscope and photographs were taken with an adapted RRID 18 HD digital camera and a Canon EOS 90D. Illustrations were imported into Adobe Photoshop 7.0 for labelling and plate composition.

### ﻿DNA extraction and sequencing

DNA was extracted from whole bodies of adults using Qiagen DNeasy Blood & Tissue Kit. A fragment of 658 bp of the cytochrome c oxidase I (COI) gene was amplified using universal barcoding primers LCO1490 and HCO2198 (Folmer et al. 1994). PCR amplification was done in a 25 μL volume using the following thermocycling protocol: 2 min at 95 °C; 5 cycles of 40 secs at 94 °C, 40 secs at 45 °C, 1 min at 72 °C; 35 cycles of 40 secs at 94 °C, 40 secs at 51 °C, 1 min at 72 °C; 5 min at 72 °C; held at 4 °C. PCR products were checked in 1% agarose gels and purified by adding 0.5 μL (10 u) Exonuclease I (Exo I) and 1 μL (1 u) Shrimp Alkaline Phosphatase (SAP). Samples were incubated at 37 °C for 15 min and reaction was stopped by heating the mixture at 85 °C for 15 min. Both strands of each fragment were sequenced using Sanger technology in Macrogen Inc. sequencing service (South Korea) with the same primers used for PCR amplification. Sequence quality check, primer trimming, and alignment were performed on CodonCode Aligner version 10.0.2. Sequences were deposited in GenBank after running a BLAST search to check for possible contaminations.

### ﻿Biology

Tests were performed inside rearing chambers (25 °C, 12 hrs. light/12 hrs. dark). Mature couples (*n* = 10) were collected from the colony and placed individually in rectangular plastic containers (20×15 cm) with fine pierced lids. Inside, a 10-cm long stem of L.g.subsp.hexapetala with a water pic was exposed for 24 hrs to each couple (Fig. 1B). Only brachypterous females were used for this test since macropterous females might have different reproductive behaviour. After the exposure, the number of oviposition scars per stem were recorded and plants were stored again in the rearing chamber until nymph emergence. Development time of eggs and the number of emerged nymphs per stem after 24 hrs of exposure were recorded. Also, laboratory-reared plants were dissected to quantify the number of eggs per oviposition scars.

To follow the developmental time of the immature stages, newly emerged nymphs (*n* = 50) were randomly selected from the previously mentioned containers and placed individually in Petri dishes with 5-cm long stems and wet tissue paper (Fig. 1C). Nymphs were observed every 1–2 days and developmental time for each instar was recorded by checking for exuviae. All means are reported with ±SD.

To estimate the host range, no-choice tests were performed in a greenhouse with controlled temperatures (26 °C day/24 °C night) and natural light conditions (summer). Because the planthopper is a sap-feeding insect, whole plants were utilised. Depending on the plant species life form, two different devices were used. If the plant was terrestrial or did not resist/withstand stems being cut to be enclosed in small containers, hanging acetate cylinders with nylon mesh on both ends were placed containing ~ 15 cm of a stem (Fig. 1D). Each cylinder was placed on independent plants. If plants were aquatic, then a single 15-cm long stem was placed inside a 3-lt plastic container with water and polyester gauze as a lid (Fig. 1A). For each plant species, between three and five repetitions were carried out, depending on plant availability. Inside each device, one male and one brachypterous female of *P.paraguayensis* were released to test survival and oviposition. For survival, the number of days elapsed from the beginning of the experiment until the death event of both males and females, were recorded. Two types of feeding controls were set up: one with the insects on the known host plant, *L.g*. subsp. hexapetala as a positive control, and another with insects only with wet tissue paper as a negative control with no food. The experiment was terminated at 52 days. For the test plant list, nine *Ludwigia* species were considered, six from the *Jussiaea* section, to which the main host plant belongs (L.grandiflora(Michx.)Greuter & Burdetsubsp.grandiflora, L.g.subsp.hexapetala, L.peploides(Kunth)P. H. Ravensubsp.peploides, L.p.subsp.montevidensis (Spreng.) P. H. Raven, Ludwigiapeploides(Kunth)P. H. Ravensubsp.glabrescens (O. Kuntze) P. H. Raven and *Ludwigiahookeri* (Micheli) H. Hara; two from the *Macrocarpon* section, *Ludwigiabonariensis* (Micheli) Hara and *Ludwigianeograndiflora* (Munz) Hara; and one from the *Myrtocarpus* section, *Ludwigia serícea* (Cambess.) Hara. Two other species, one closely related species from another genus of Onagraceae, *Oenotheraaffinis* Cambess. and one that is not closely related but commonly co-exist in the natural habitat, *Myriophyllumaquaticum* (Vell.) Verdc were also evaluated.

### ﻿Data analysis

Survival data of females was analysed through Kaplan-Meier survival curves using the ‘survfit’ and ‘ggsurvplot’ functions from Survival package (ver. 3.4-0) of RStudio statistical program (ver. 2023.06.1). A non-parametric Log-rank statistical test was used to assess statistical differences in overall survival. To better understand the influence of each test plant in *P.paraguayensis* survival, the Cox regression model was employed to estimate the hazard ratio (HR) and *P* values based on χ^2^ test, and 95% confidence intervals (95%-CI). HR is the rate of occurrence of an event during a given time interval, where HR > 1 means that exposure to the factor increases the rate of occurrence of the event, and HR < 1 decreases the rate. If the HR = 1 we say that the factor behaves as the reference (Kleinbaum and Klein 2012). Forest Plot for Cox proportional hazards model was created using the ‘coxph´ and ‘ggforest’ functions of R packages survival and survminer (0.4.9). P < 0.05 defined significance. All analyses were conducted with R 4.3.1 (R Core Team 2022).

## ﻿Results

### ﻿Morphology of *Pissonotusparaguayensis* Bartlett, 2000: 144

**Adult**

This species was described in detail by the author from a brachypterous male from Paraguay; the brachypterous female was briefly discussed, referring only to its body size with some observations on the variations in colour patterns among specimens from different geographic locations (Bartlett and Deitz 2000).

**Figure 2.**
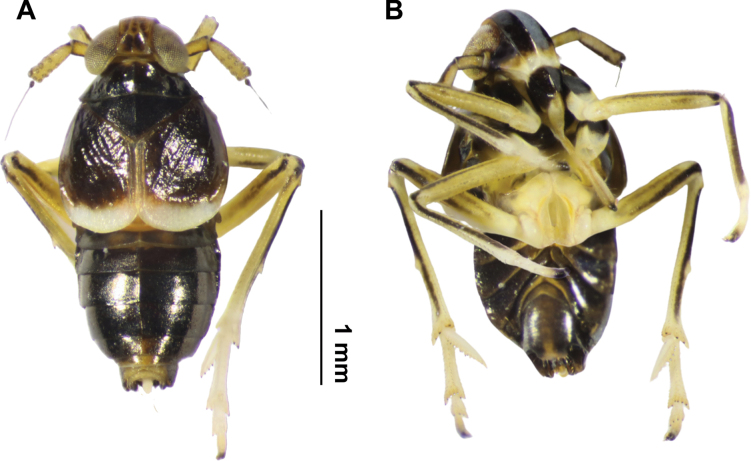
*Pissonotusparaguayensis*. Habitus. Brachypter male **A** dorsal view **B** ventral view.

**Male (Fig. 2).** Although the brachypterous male has been fully described and illustrated by the author, we added representative images of that morph in order to facilitate its association and recognition, given the dimorphic sexual coloration of this species.

**Figure 3.**
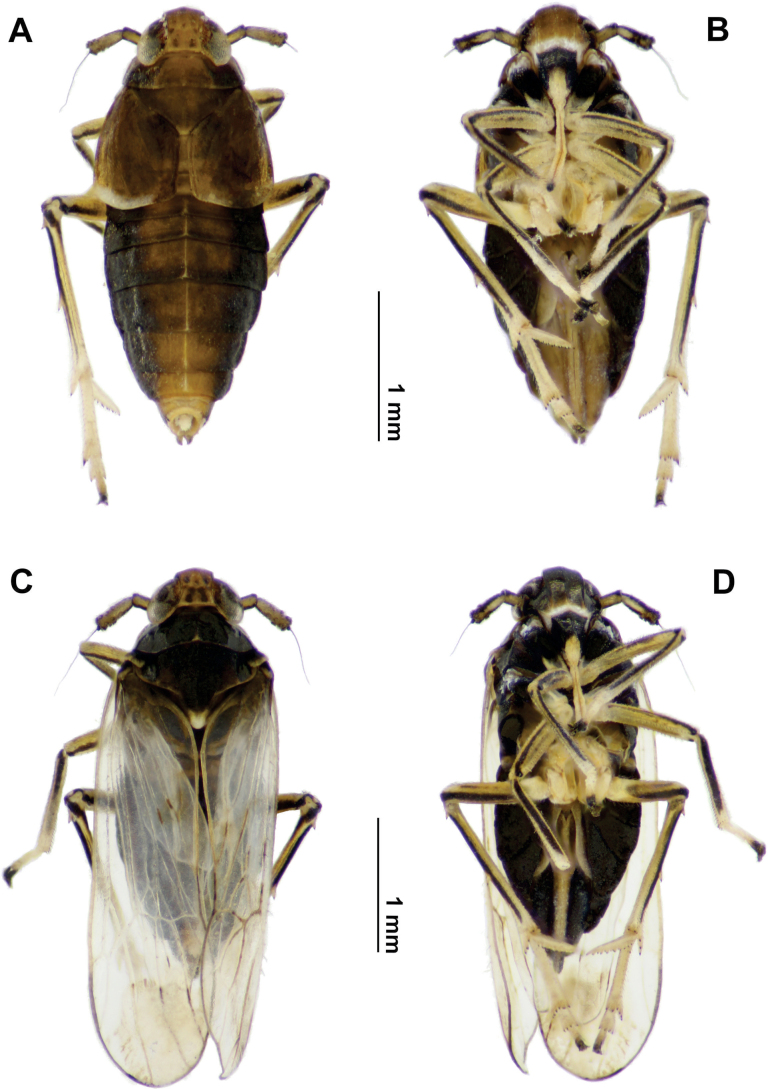
*Pissonotusparaguayensis*. Habitus. Brachypter female **A** dorsal view **B** ventral view. Macropter female **C** dorsal view **D** ventral view.

**Figure 4.**
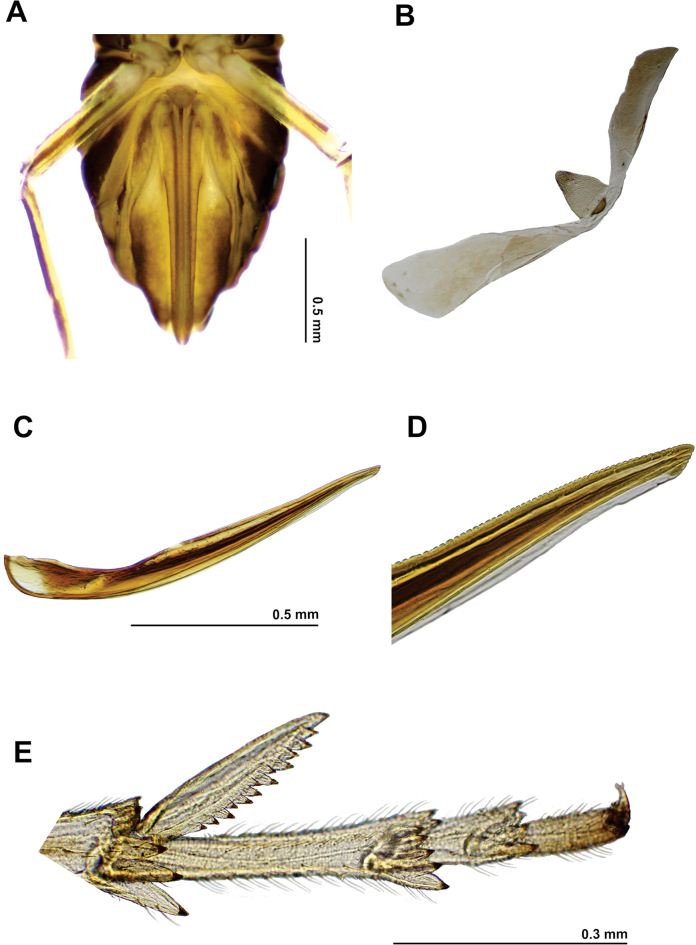
*Pissonotusparaguayensis* female **A** abdomen, genital region, ventral view **B** pregenital sternite **C** gonapophysis IX **D** apex of gonapophysis IX **E** apex of hind leg (post tibial spur and tarsi) of adults.

**Female (Figs 3, 4).** Note: As Bartlett and Deitz (2000) noted, females of *Pissonotus* have been little used in taxonomy probably due to the scarce availability of specimens reliably associated with males. Illustrations and complementary comments not included in the original description, particularly on variation in colour between or within conspecific populations and descriptions of some structural features of the female genitalia are given for the first time.

**Brachypter.** Body length: 2.91–3.16 (3.01, *n* = 5); tegmina length: 0.80–0.90 (0.82, *n* = 4) (Fig. 3A, B).

***Colouration***: In dorsal view body generally dark brown, paler on vertex and along midline of abdomen and widening above genital and anal segments. Vertex and upper frons are mostly brownish with carinae and anterior margin paler. Tegmina dark brown with a distinguishable distal transverse narrow white band, sometimes medially incomplete; veins concolourous with venation obscure becoming obsolete toward apex (not reticulate). In ventral view, head with a distinctive transverse whitish band on epistomal margin of frons, which extends laterally along the genae to the base of the pre- and mesocoxae; blackish brown area over postclypeus and basal ½ of coxae contrasting with the yellowish colouration of anteclypeus, rostrum, thoracic sternites and legs. Apex of rostrum, longitudinal anterior highlights on antennomeres I and II and dorsal margins of femora and tibiae, and the apex of last tarsal segments and ungues, black. Abdomen paler along midventral line from sternum V to the caudal region.

***Structure***: Antennal foveae elevated, placed close to ventral margin of the compound eye. Tegmina quite longer as broad (6:1), distally rounded on leading margin and apically truncate, not exceeding tergite II. Calcar slender, moderately foliaceous; shorter than basitarsus (0.8:1), usually with a row of 13–15 black tipped teeth on ventral margin with 2–4 smaller teeth irregularly spaced external to the row (Fig. 4E).

**Macropter.** Total length (including wings): 3.66–3.70 (3.68, *n* = 4). Body length (excluding wings): 3.00 (3.00, *n* = 4), fore-wings length: 2.90 (*n* = 4). (Fig. 3C, D).

***Colouration***: Similar to brachypterous form in colour pattern of head, antennae, sternothorax, and legs. General body colouration uniformly darker; with distinctive yellowish areas: dorsally, restricted on caudal apex of mesonotum, base of tegula and anal segment, and ventrally, on the posterior margin of abdominal sternites, pregenital sternite, inner margins of gonocoxa VIII and gonapophysis VIII. Wings semi-hyaline, fore-wing veins light brown, proximal portion of remigium and clavus and some longitudinal and transverse veins, quite dark.

***Structure***: Similar to the brachypterous form, with mesothorax more robust. Wings fully developed, longer than abdomen, fore-wings extending ~ 1/3 of their length beyond the tip; hind-wings shorter. Fore-wing with a simple venation pattern, with scattered small seta-bearing tubercles irregularly distributed on the veins. Remigium veins mainly non-bifurcated, nodal cells poorly defined, sometimes absent, with four long postnodal cells distally open, CuP apically weakened; pre and post nodal cells elongated; clavus in angle, postclaval margin forming an obtuse angle (100–120°).

***Genitalia*** (Fig. 4A–D): Ovipositor sclerotised and relatively long and narrow, shortly surpassing the posterior margin of anal segment. Pregenital sternite subtriangular, elongate, longer in middle line than its wide at its widest part (1.5:1), anterior margin strongly narrowed “like a neck” and lightly rounded at apex (Fig. 4A, B). Gonocoxa VIII, more or less lanceolate, regularly expanded at base; as long as 1/3 the length of the gonapophyses VIII (Gy VIII) which is uniformly slender and elongated. Gonapophyses IX slender, slightly curved, wider at basal 1/3, distal 1/3 with minute teeth on dorsal margin (Fig. 4C, D); dorsal gonoplac (Gp) or third valvula uniformly wide. Anal style moderately short.

**Host Plant.** In the field, adults and nymphs were observed feeding on Ludwigiagrandiflorasubsp.hexapetala.

**Recorded natural enemies.** None.

**Distribution.** South Central South America: Brazil, Paraguay and apparently Bolivia (Bartlett and Deitz 2000). Argentina: Santa Fé, Entre Ríos and Buenos Aires.

*Pissonotusparaguayensis* was found in wetlands of central Argentina that belong to the Del Plata Basin, expanding its geographic range to central-east of Argentina and now making Buenos Aires province its southernmost limit of distribution in America (Table 1).

**DNA barcode.** The two sequenced specimens (1♀b, 1♂b) from Brazo Largo, Entre Ríos (33°51'53.4"S, 58°52'59.4"W) had the same 658 bp COI haplotype, which was deposited in GenBank under accession number OR523788.

**Material examined.** Argentina: 6♀b, 1♀m, Buenos Aires, Otamendi, 34°13'04"S, 58°55'52"W, s/ L.g.subsp.hexapetala, 21-01-2019, Faltlhauser col.; 10♂, same place and host, 10-03-2019, Hernández col.; 15 ♂, same place and host, 07-06-2019, Hernández col.; 1 ♀m, 8-10-2008, (named 1 ♀ Delphacidae), Cabrera Walsh col.; 15♂, 3♀b, laboratory reared (FuEDEI), 7-06-2019, Hernández; 5♀m, 2♀b, 1♂b, Buenos Aires, Magdalena, 35°3'48.9"S, 57°33'14.9"W, s/ L.g.subsp.hexapetala, 3-11-2021, Faltlhauser col.

**Remarks.** Although the colour pattern is useful to recognise members of the genus, some variations were observed among brachypterous specimens of both sexes. General colouration varies from black to blackish-orange, particularly the distal transverse white band of tegmina that may be complete, medially incomplete, or exceptionally absent, and the dorsal surface of the abdomen with more diffuse colouration towards the caudal region. Nevertheless, the distinctive pattern colouration described on the head and thorax is rather uniform in both sexes and morphs. The drumming organ in males shows a dimorphic distinctive yellowish colouration. Macropterous forms show more homogeneous colouration among specimens.

**Figure 5.**
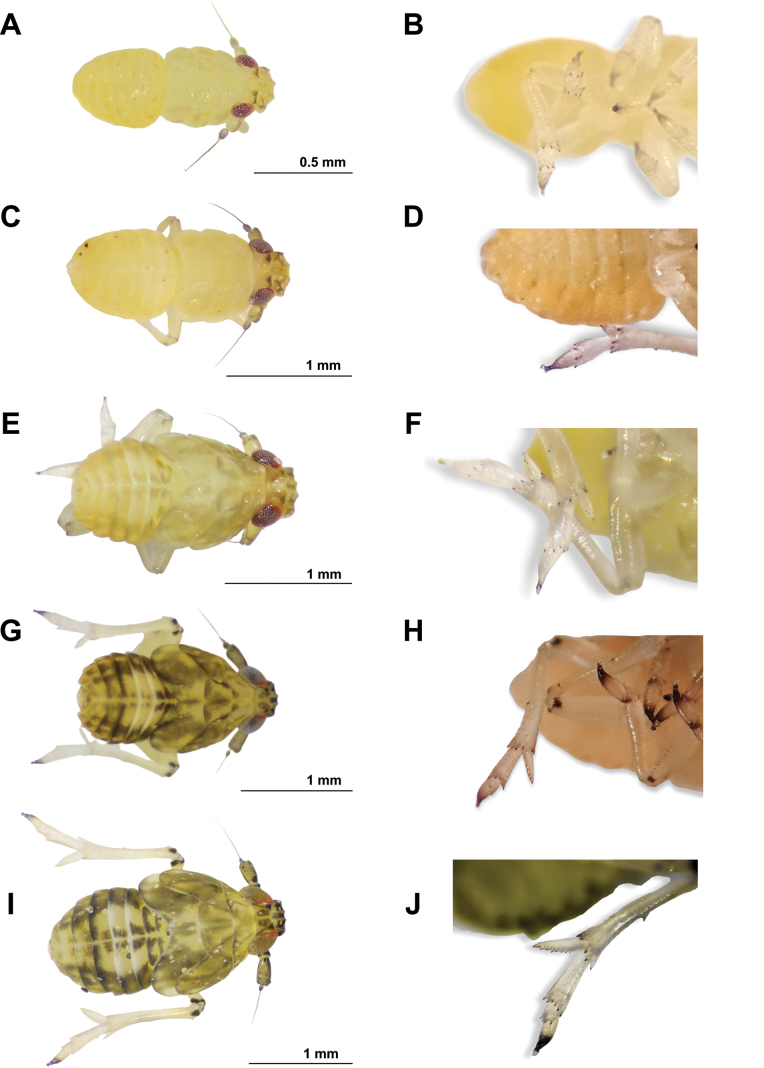
Immature stages in dorsal view of *Pissonotusparaguayensis***A** first instar **C** second instar **E** third instar **G** fourth instar **I** fifth instar (presumptive brachypter form). Apical portion (from tibiae) of hind leg **B** first instar **D** second instar **F** third instar **H** fourth instar **J** fifth instar (presumptive brachypter form). Scale bars: 0.5 mm (**A**); 1mm (**C**, **E**, **G. I**).

**Immature stages.** Fig. 5.

### ﻿Key to the instars of *Pissonotusparaguayensis*

**Table:** 

1	Metatarsi two-segmented. General colour pale, uniform yellow on dorsal view	**2**
–	Metatarsi tri-segmented, or metatarsomere 2 partially subdivided in the middle. General colour grey to pale yellow dorsally mottled dark brown and cream	**4**
2	Body uniformly yellow, antenna uniformly grey. Metatibia without lateral spines, spur < 2× length of the longest apical spines of the metatibia. Antennal pedicel without sensorial pits (Fig. 5A, B)	**First instar**
–	Body yellow with a dark marking on upper frons, antenna, apex of rostrum, femoro-tibial joins, dorsal pro and mesotarsite, apex of tarsites and ungues. Metatibia with lateral spines, spur > 2× the length of the longest apical spine of the metatibia. Antennal pedicel with sensorial pits	**3**
3	Metatibial spur without marginal teeth. Metatarsomere 1 with a transverse apical row of 4 spines (Fig. 5C, D)	**Second instar**
–	Metatibial spur with ≥ 2 marginal teeth. Metatarsomere 1 with a transverse apical row of 5 spines (Fig. 5F)	**4**
4	Metatibial spur with two or three marginal teeth. Wing pads weakly developed, barely projecting laterally (Fig. 5E, F)	**Third instar**
–	Metatibial spur with ≥ 3 marginal teeth. Wing pads developed	**5**
5	Metatibial spur with 4 or 5 marginal teeth. Fore-wing pads laterally reaching the middle of hind-wing pad (Fig. 5G, H)	**Fourth instar**
–	Metatibial spur with 7–9 marginal teeth. Fore-wing pads covering lateral 2/3 of hind wing pads; hind-wing pads not reaching third tergite (Fig. 5I, J) (brachypterous form) or extending near apex of hind wing pads, overlapping third tergite (Fig. 6A, B) (macropterous form)	**Fifth instar**

**Description: Eggs.** (Fig. 7A) (*n* = 10). Length: 0.86, width: 0.23.

Eggs milky white, ellipsoidal. Chorion translucent.

**First Instar.** (Fig. 5A, B) (*n* = 5). L:1.04 (0.93–1.07) W: 0.33 (0.29–0.36); t.l: 0.35 (0.25–0.41).

Uniform whitish body; with distinctive red eyes; uniform greyish antennal segments.

Form elongate, subcylindrical, dorsoventrally slightly flattened. Vertex subquadrate, with lateral margins often divergent in front and behind the eyes; length sub-equal to width (1.2:1); fastigium (lateral view) rounded; lateral and submedian carinae of vertex extending onto frons. Frons with lateral margin slightly convex and carinate, ~ 2× longer than wide in midline, regularly wider towards the frontoclypeal suture; lateral margins carinate and paralleled by submedian carina which is prominent at base and regularly evanescent towards apex; each laterofrons at level below the eyes 1.5× wider than interfrons; with 13 sensorial pits on each side: nine pits on area between submedian and lateral carinae (six visible in ventral view, three in dorsal view); dorsally four pits between lateral carina and eyes. Antennae three-segmented; scape, short, slightly wider than long, antennal pedicel sub-cylindrical ~ 2–2.5× the length of scape, without sensory pits; base of flagellum bulbous ~ 0.5× the pedicel length. Rostrum reaching the posterior margin of metacoxae, apical segment 0.6× longer than subapical.

Thoracic nota divided into three pairs of plates by longitudinal mid-dorsal line. Pronotal plates subtrapezoidal, lateral carinae divergent and slightly convex towards posterior margin. Each plate with seven pits extending from near middorsal line posterolaterally to lateral margin (two pits in line next to lateral carinae on posterior ½ of segment and five pits extending laterally from the external side of carina along the posterior border of plate, one proximal to it and four most lateral not visible in dorsal view). Mesonotum as long as metanotum. Mesonotal plate with four pits, two median on disk, and two next to lateral margin. Pro, meso, and metatarsi with two tarsomeres; apical tarsomeres subconical, curved, with a pair of apical claws. Metatibiae without lateral spines, with apical transverse row of four stiff spines; tiny spur < 2× the length of longest apical spines of the metatibia and ~ 1/4 length of metatarsomere 1. Metatarsomere 1 shorter than metatarsomere 2 (0.5:1) with four apical spines.

Abdomen with nine apparent segments, widest across segment 5. Tergite 5 with one pit and tergites 6–8 each with three pits on either side of the midline. Segment 9 surrounds anus with three pits on each side, two subapical and one dorsal median.

**Second Instar.** (Fig. 5C, D) (*n* = 5). L: 1.57 (1.54–161); W: 0.61 (0.61–0,73); t.l.: 0.48 (0.44–0.52).

Pale yellowish body, with distinctive black markings on the head and legs: a stripe on apex of vertex and upper frons extended at lower level of the eyes, along the frontal surface of antennal segments I and II, and several marks limited to: upper border of antennal fovea, apex of rostrum, outside apices of femoro-tibial joints, base of basitarsus and apices of legs.

Antennal foveae elevated, placed under the compound eyes, close to its lower margin; antennal pedicel sub-cylindrical ~ 3× the length of scape, with three small pits: two on apical margin and the third subapical on posterior surface; flagellum whip-like distally, bulbous at base, ~ 0.25× the pedicel length. Rostrum extends almost to bases of metacoxae, apical segment 0.2× shorter than subapical. Pro, meso, and metanotum subequal, pronotum slightly shorter (0.7:1); posterior margin of mesonotum straight at middle, metanotum deeply excavated. Wing-pads undeveloped. Metatibiae with two small lateral spines (one near base and one in basal ½), and five stiff spines on the apical transversal row; spur ~ 1/4 length of metatarsomere 1, with two similar sized teeth, one on lateral aspect added to the apical. Metatarsomere 1 as long as metatarsomere 2, with apical transversal row of five spines.

**Third instar.** (Fig. 5E, F) (*n* = 4). L: 1.78 (1.62–1.81); W: 0.89 (0.85–0.93)); t. l.: 0.56 (0.50–0.62).

Similar pattern colour to second instar, with darker markings.

Antennal pedicel sub-cylindrical, ~ 3× the length of scape, with four to five sensory pits; flagellum with bulbous portion ~ 0.1 the pedicel length. Rostrum overlapping the bases of metacoxae, apical segment 0.3× shorter than subapical.

Mesonotal plate with the two discal pits on both sides of the lateral carinae. Fore-wing pads short, each covering 1/2 of metanotal segment laterally, with three pits, a pair on costal area and another near middle. Metanotum plates median length as long as mesonotum, shallowly excavated on posterior margin. Metatrochanter subcylindrical, with row of ten or eleven interlocking flattened folds on posteromedial aspect. Metatibial spur 3/4 the length of metatarsomere 1, with one apical tooth and two to three teeth on lateral margin. Metatarsomere 1 with apical transverse row of five spines; apical tarsomere with an arrange of fine sensory hairs along it.

**Fourth instar.** (Fig. 5G, H) (*n* = 10). L: 1.80 (1.69–1.89); W: 0.87 (0.73–1.00); t. l: 0.55 (0.51–0.56).

Colour pattern similar to former instar. Most specimens with conspicuous brownish marking along dorsal portions of thoracic nota continuing onto wings pads and abdomen.

Antennal pedicel sub-cylindrical ~ 2× the length of scape, with approx. five or six pits on the apical ½ (Fig. 5B). Rostrum extending to bases of metacoxae, apical segment slightly shorter than subapical.

Fore-wing pads short, each covering ~ 1/2 of hind wing pad, laterally with three pits, one on costal area and two next to subcostal carina. Metanotum median length sub-equal to mesonotum; hind-wing pad oval, reaching the base of the first abdominal segment. Metatibial spur almost as long as the metatarsomere 1 in middle, with one apical tooth and three or four on lateral margin. Metatarsi with two tarsomeres: tarsomere 1 with apical transverse row of six spines, tarsomere 2 subconical, similar to apical tarsomeres of other legs, slightly shorter than length of tarsomere 1, partially subdivided in middle, with three short ventrolateral spines in middle of plantar surface.

**Figure 6.**
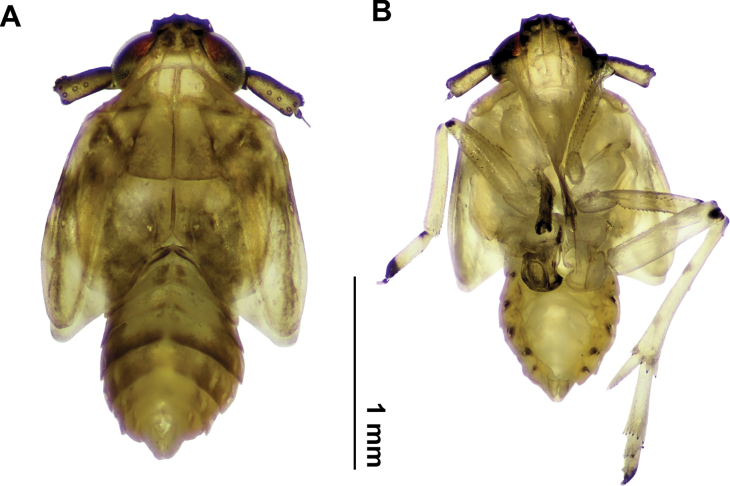
Fifth instar (presumptive macropterous form) *Pissonotusparaguayensis***A** dorsal view **B** ventral view.

**Fifth instar.** Presumptive brachypterous form (Fig. 5I, J) (*n* = 10) L: 2.27 (2.18–2.36); W: 0.99 (0.96–1.01); t. l: 0.65 (0.62–0.70). Presumptive macropterous form (Fig. 6A, B) (*n* = 2). L: 2.3; W: 1; t.l; 0.9.

Colour pattern similar to former instars but darker. Dorsally blackish mottled with light to dark brown and cream on head, thorax and abdomen, with notorious yellowish pits; ventrally, head with postclypeus also darkened at base. Obvious black mark on legs: on base of coxae, outside apices of femoro-tibial joint, longitudinal stripes on dorsal margins of femora and tibiae, basal tarsomere of pro and mesotarsi and the apex of last tarsal segments and claws.

Form elongate, sub-cylindrical, widest across mesothoracic wing pads. Head lightly protruding beyond the anterior margin of eyes, ~1/4 of the eyes length. Vertex subquadrate, length slightly longer than wide (1:07), anterior margin straight, posterior convex, basal compartments shallowly concave; lateral and submedian carinae prominent, continuing onto frons from the level of middle of the eyes to clypeal margin. Frons sub-rectangular; widest at middle, width. ~ 0.9× the length; carinate lateral margins slightly convex, these lateral carinae extending from vertex to clypeal border and paralleled by a pair of straighter submedian carinae regularly evanescent towards clypeal border. Clypeus narrowing distally, consisting of subconical basal postclypeus not carinated along its length, and cylindrical distal anteclypeus. Antennae with scape cylindrical, length subequal to width; pedicel subcylindrical, ~ 3× longer than scape, with nine or ten pits on the apical ½. Rostrum surpassing mesocoxae, apical segment slightly longer than apical (1.2:1).

Pronotal plates sub rectangular; anterior margin follows posterior margin of head, posterior border almost straight; each plate with straight short posterolaterally directed carina originating on anterior margin in median 1/3 and terminating near middle of plate, carina bordered along inner margin by row of six pits extending posterolaterally to lateral border of plate (three pits in line next to lateral carinae and three near posterior margin; one lateralmost pits not visible in dorsal view). Mesonotal median length ~ 1.5× that of pronotum; each plate bearing an elongate lobate wingpad covering lateral ½ of metanotal wingpad; with posterolaterally directed carina originating on anterior margin in median 1⁄4 and terminating on posterior margin, with five pits: two on notum, one on each side of carina, and three laterad (two on wingpad). Metanotal median length ~ 0.75× that of mesonotum; each plate bearing an elongate lobate wingpad extending to tergite 2; with weak longitudinal carina originating on anterior margin in median terminating near posterior margin; one pit on wingpad, just lateral to carina. When developing to macropterous adults the fore-wing pads extend up to the apices of the hind pads, reaching tergite 5.

Pro- and mesocoxae elongate, posteromedially directed; metacoxae fused to sternum. Metatrochanter subcylindrical, with a row of 12–14 flattened folds on the posteromedial aspect which interlocks with those on the adjoining trochanter. Metatibiae with subtriangular, slender, moderately foliaceous spur, a little shorter than metatarsomere 1, with a row of seven-nine teeth on lateral margin and one apical tooth. Metatarsomere 1, longer than tarsomere 2 plus 3 (1:05), with apical transverse row of seven black-tipped spines on plantar surface; tarsomere 2 cylindrical, with apical transverse row of four black-tipped spines on plantar surface; tarsomere 3 sub conical, similar to apical tarsomere of other legs.

Abdomen with nine apparent segments, slightly flattened dorsoventrally, widest across segment 5. Segment 9 surrounds anus with three evident pits on each side, two subapical and one dorsal median, in both sexes.

**Specimens examined.** Argentina: Otamendi, Buenos Aires (34°03'48.2"S, 58°49'19.59"W), 10 nymphs V, 10 nymphs IV, 10 nymphs III, 10 nymphs II and 10 nymphs I, 07-06-2019, on L.g.subsp.hexapetala, Faltlhauser col.; 2 nymphs III, 1 nymph I; 21-I-19, on L.g.subsp.hexapetala, Faltlhauser col.

### ﻿Biology

#### Field observations

**Figure 7.**
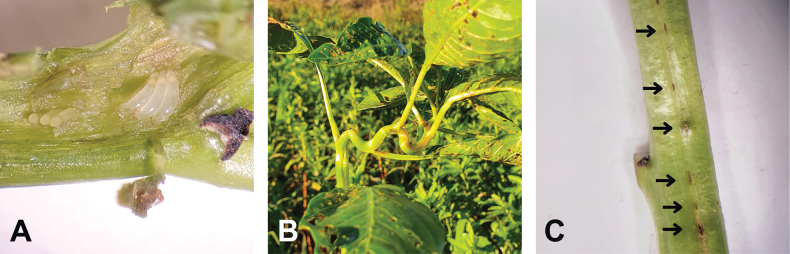
**A***Pissonotusparaguayensis* eggs oviposited in Ludwigiagrandiflorasubsp.hexapetala**B** damage observed on L.g.subsp.hexapetala produced by ovipositions **C** oviposition marks.

In this region, the early months of summer are not the most suitable to find this planthopper. A peak of abundance in the Buenos Aires region was observed between March and July. In the field, they were found on Ludwigiagrandiflorasubsp.hexapetala, normally higher up on the plant. Adults and nymphs tended to move to the underside of the leaves and stems or hop to the water surface when disturbed. Adults were more often found on glabrescent stems. The insertion of eggs on the plant stems produces a corkscrew distortion damage that can be easily observed (Fig. 7B).

#### Life history

The female of *Pissonotusparaguayensis* inserts its eggs in the plant tissue on either side of the leaf and in the upper part of the stems further away from the water surface (Fig. 7A-C). After 24 hrs of exposure to *L.g*. subsp. hexapetala, brachypterous females produced an average of 18.13 ± 3.68 oviposition scars (range 13–23; *n* = 10, hereafter means are reported with ±SD) in a 10-cm stem at 25 °C. Each oviposition scar had a mean number of 2.3 ± 0.82 eggs (range 1–3; *n* = 10) (Fig. 7C). The mean development time from oviposition to hatch was 10.6 ± 0.9 days (range 9–12; *n* = 214). Generation time (egg to adult) was 36.71 ± 1.98 days (range 33–41; *n* = 21). The development time for nymphs I, II, III, IV, and V were 6.7 ± 0.5 days (range 6–8; *n* = 36), 3.2 ± 0.7 days (range 2–6, *n* = 35), 4.6 ± 1.0 (range 3–7; *n* = 31), 4.1 ± 1.2 days (range 2–6; *n* = 25), 7.1 ± 1.6 days (range 5–11, *n* = 21) respectively. Female adults can live over 50 days (*n* = 30) in laboratory conditions.

#### Host range

**Figure 8.**
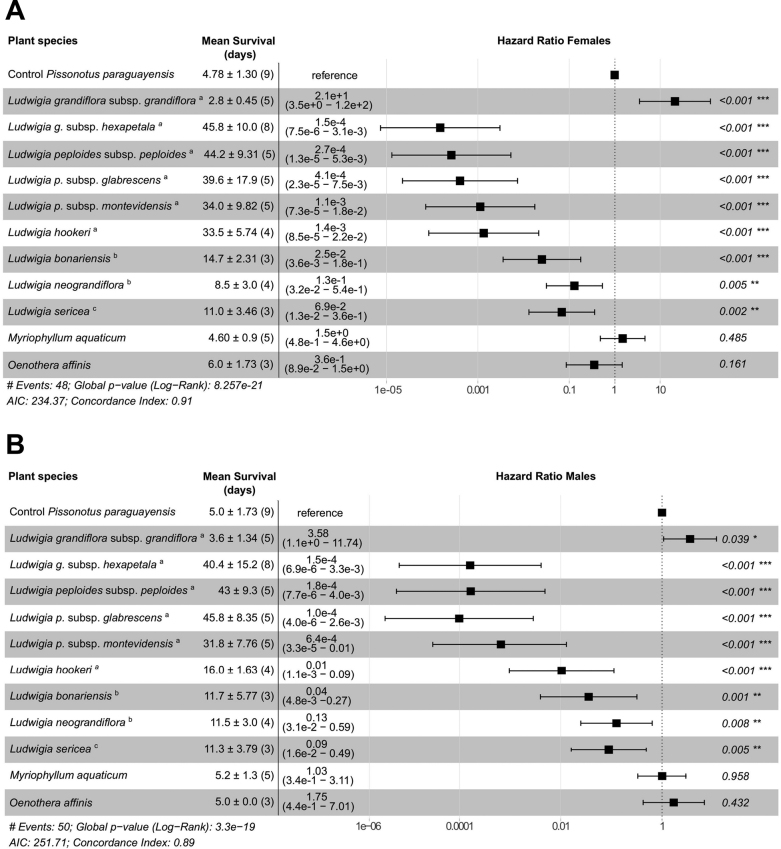
Forest plot of Cox proportional hazard analysis for *Pissonotusparaguayensis***A** females and **B** males survival where hazard ratio (HR) > 1 means that exposure to the factor increases the rate of occurrence of the event (cavity lost), and HR < 1 decreases the rate. If the HR = 1 the factor does not influence survival (Kleinbaum and Klein 2012). HR are depicted by box symbols with confidence bands and parenthetical values representing 95% confidence intervals. The p values represent Wald test of significance and magnitude of significance is denoted with asterisks (*). AIC, Akaike information criterion. Small letters next to plant species indicate sectional classification of *Ludwigia*: ^a^*Jussiaea*, ^b^*Macrocarpon*, ^c^*Myrtocarpus* based on Raven (1963) and Wagner et al. (2007).

This species of delphacid was found in the field on L.g.subsp.hexapetala. However, survival and host range revealed that *P.paraguayensis* was able to survive in other congeneric species to the known host plant. Through the Kaplan-Meier analysis, we observed that *P.paraguayensis* had better survival probability for species inside the Jussiaea section except for L.g.subsp.grandiflora. Log-Rank tests determined that there were significant median differences in the survival probability between plant species, both for females and males (χ^2^ = 117, 11 df, p = < 2e^-16^; χ^2^ = 93.3, 11 df, p = 4e^-15^, respectively (Suppl. material 1)). Cox proportional hazard analysis revealed that both males and females of *P.paraguayensis* behaved similarly (Fig. 8A, B). *P.paraguayensis* survived an average of five days without food, only water, and the same performance was observed when offered *M.aquaticum* and *O.affinis*. There were no significant differences between species inside the *Jussiaea* section, surviving the longest in L.g.subsp.hexapetala (45.8 ± 10.0 days). However, Ludwigiag.subsp.grandiflora was detrimental to survival, dying even sooner than the control. Survival in species of other *Ludwigia* sections was significantly lower (HR closer to 1) and are not considered suitable hosts.

## ﻿Discussion

This study contributes to the actualisation of *Pissonotusparaguayensis* by describing adult females (brachypterous and macropterous) and immature stages for the first time. Although the female genitalia had not been described or used in *Pissonotus* systematics, the combination of pregenital sternite and ovipositor morphology of *P.paraguayensis* is here proposed as a new complementary diagnostic trait for the delimitation of the genus and its species. However, future studies will allow us to analyse its importance. Photographs of *P.marginatus* Van Duzee female available in Bartlett (2020) show the presence of the pregenital sternite although it did not receive any mention by the author.

Macropterous females, previously unknown for the species, show the venation pattern quite simplified, which could correspond to the brachypterism pattern described by Bourgoin et al. (2015). DNA-based taxonomic methods can help clarify species relationships within this group as well as aid in the identification of both adult and immature stages. The obtained COI sequence for *P.paraguayensis* is the first one recorded for the genus in South America and joins 14 other species for which sequences are available on GenBank and BOLD databases at the time of writing (7 Sept 2023). Of the 43 known species of the *Pissonotus* genus, only the North American *P.delicatus* had the immature stages described (Bartlett and Deitz 2000). Based on the Wilson and Tsai (1991) description, fifth instar nymphs of *P.paraguayensis* differ from the latter mainly by the pattern colouration: blackish colouration on dorsal of head, thorax and abdomen with conspicuous yellowish pits, ventrally only darkened on base of frons extended to lower level of the eyes and dorsal surface of antennomeres I and II, and legs with distinctive black marks on femoro-tibial join and apex.

Immature stages of *P.paraguayensis* can be distinguished from other three species of delphacids that inhabit and feed on aquatic macrophytes in Argentina, *Megamelusscutellaris* Berg (Sosa et al. 2005), *M.bellicus* Remes Lenicov and Sosa (Mariani et al. 2007) and *Lepidelphaxpistiae* Remes Lenicov (Marino de Remes Lenicov et al. 2017), by a combination of morphological and colouration features. These main features are the yellowish colouration without defined stripes on frontoclypeal area, distinctive blackish marks on the upper front of the head, basal antennal segments, thorax, legs and abdomen; also the slender spur has eight subequal marginal fine black tipped teeth.

The previous distribution range reported for *P.paraguayensis* was Paraguay, Bolivia, and Brazil (Bartlett and Deitz 2000). This work provides an updated distribution, expanding its range for the first time into Argentina, being Buenos Aires the southernmost limit of the genus (35°3'48.9"S, 57°33'14.9"W). Moreover, the aquatic invasive plant, L.g.subsp.hexapetala is recorded as the only known host plant in natural conditions.

Little information is known on the ecology of the genus. Records from Buenos Aires province indicate that *P.paraguayensis* was more frequently observed in autumn months, specifically from March to July. This differs from the recorded patterns for the South American species *P.boliviensis* and *P.neotropicus*, which are primarily observed between January and April, as reported by Bartlett and Deitz (2000). In the field, *P.paraguayensis* can be indirectly detected by observing a corkscrew or wavy distortion of the L.g.subsp.hexapetala stems produced by the damage of the egg insertions.

Although there are no natural enemies recorded for this species, members of the Order Hymenoptera (Dryinidae and Mymaridae) and Strepsiptera have been reported as parasitoids of adults and nymphs of three North American species (adults of *P.dorsalis* (Van Duzee), third instar of *P.delicatus* and eggs of *P.quadripustulatus* (Van Duzee)) (Stiling and Moon 2005). Bartlett (2020) (and updates) mentioned *Anagrusflaveolus* Waterhouse (Mymaridae) as an egg parasite of *Pissonotus* sp. and an undetermined Dryinidae on probably *Pissonotusconcolor* Bartlett.

Summarising information on the species biology and host plants, host range tests showed that *P.paraguayensis* presents a certain degree of specificity to the Jussiaea section of the genus Ludwigia and that therefore it can survive not only in L.g.subsp.hexapetala. Even though L.g.subsp.grandiflora is closely related to the main host plant, it was the only species inside the section where *P.paraguayensis* did not survive. Stems of this species are covered by dense pubescence of soft, viscid hairs, sticky when fresh, which usually trap small insects impeding any further movements. The results obtained are similar to others observed in the search for other control agents for L.g.subsp.hexapetala (Reddy et al. 2021; DaSilva et al. 2022). The species of the section Jussiaea form a distinctive, closely related group, especially due to the apparent lack of genetic barriers between entities of this group (Zardini et al. 1991). Nearly all species of this section can be crossed with one another and produce vigorous F1 hybrids (Peng 1990); therefore, finding a control agent specific to one of those species has been proven to be difficult. However, *P.paraguayensis* did not survive in the other *Ludwigia* species tested outside *Jussiaea*. There are successful records of delphacids as biological control agents against invasive aquatic plants, *Prokelisiamarginata* (Van Duzee) to control *Spartinaalterniflora* Loisel. in Western USA (Grevstad et al. 2003) and *M.scutellaris* to control *Pontederiacrassipes* (Mart.) Solms. in South Africa and USA (Tipping et al. 2014; Paterson et al. 2023). With the new morphological and biological knowledge about *P.paraguayensis*, it is now possible to move forward and perform more host specificity tests (e.g. multiple generations, ovipositions, field population dynamics) with larger test plant lists to establish if this planthopper can be proposed as a biological agent against *Ludwigia*.

In conclusion, the description of both forms of *P.paraguayensis* females and immature stages presented in this work, not only contributes to the species complete description but also provides new morphological and molecular tools to identify the species inside the genus. The collection of *P.paraguayensis* in Argentina has extended the distribution of the species and genus and enriched the country’s biodiversity. A new rearing methodology was developed both to establish an indoor/outdoor colony and to perform different tests in laboratory conditions to learn more about its biology and potential as a biological control agent.

## ﻿Additional information

### Author ORCIDs

Ana M. Marino de Remes Lenicov https://orcid.org/0000-0001-8678-5408

Ana C. Faltlhauser https://orcid.org/0000-0003-3121-3650

Alvaro Foieri https://orcid.org/0000-0001-5623-3629

Nicolas A. Salinas https://orcid.org/0000-0002-1055-4475

M. Cristina Hernández https://orcid.org/0000-0003-4382-773X

Alejandro J. Sosa https://orcid.org/0000-0002-1680-8712

### Data availability

All of the data that support the findings of this study are available in the main text or Supplementary Information.

## Supplementary materials

Supplementary material 1 Graphical figures of Kaplan-Meier curves of *Pissonotusparaguayensis* males and female survival probability on different test plants Data type docx Explanation note Kaplan-Meier curves of male and female *Pissonotusparaguayensis* survival probability on different test plants (colours). Non-parametric Log-rank statistical test was used to assess statistical differences in overall survival. https://binary.pensoft.net/file/963921This dataset is made available under the Open Database License (http://opendatacommons.org/licenses/odbl/1.0/). The Open Database License (ODbL) is a license agreement intended to allow users to freely share, modify, and use this Dataset while maintaining this same freedom for others, provided that the original source and author(s) are credited.Ana M. Marino de Remes Lenicov, Ana C. Faltlhauser, Alvaro Foieri, Nicolas A. Salinas, M. Cristina Hernández, Alejandro J. Sosa

## References

[B1] Asche M (1985) Zur phylogenie der Delphacidae Leach, 1815 (HomopteraCicadinaFulgoromorpha). Marburger Entomologische Publikationen 2(1–2): 1–910.

[B2] Bartlett CR (2020) [and updates] Planthoppers of North America. https://sites.udel.edu/planthoppers/ [Accessed on 01 Mar 2023]

[B3] Bartlett CR Deitz LL (2000) Revision of the New World Delphacid Planthopper Genus *Pissonotus* (Hemiptera: Fulgoroidea). Thomas Say Publications in Entomology, Entomological Society of America, 234 pp. 10.4182/AKAE8776

[B4] Bourgoin T Wang R Asche M Hoch H Soulier-Perkins A Stroiński A Yap S Szwedo J (2015) From micropterism to hyperpterism: recognition strategy and standardized homology-driven terminology of the forewing venation patterns in planthoppers (Hemiptera: Fulgoromorpha). Zoomorphology 134(1): 63–77. 10.1007/s00435-014-0243-6 25705075 PMC4326643

[B5] Chen S Yang CT (1995) The metatarsi of the Fulgoroidea (Homoptera: Auchenorrhyncha). Chinese Journal of Entomology 15: 257–269.

[B6] Crawford L (1914) A contribution toward a monograph of the homopterous insects of the family Delphacidae of North and South America. Proceedings of the United States National Museum 46(2041): 557–641. 10.5479/si.00963801.46-2041.557

[B7] DaSilva A Reddy AM Pratt PD Friedman MSH Grewell BJ Harms NE Faltlhauser AC Chamorro ML (2022) Biology of immature stages and host range characteristics of *Sudauleutesbosqi* (Coleoptera: Curculionidae), a candidate biological control agent of exotic *Ludwigia* spp. in the USA. The Florida Entomologist 105(3): 243–249. 10.1653/024.105.0310

[B8] Denno RF Roderick GK (1990) Population Biology of Planthoppers. Annual Review of Entomology 35(1): 489–520. 10.1146/annurev.en.35.010190.002421

[B9] Denno RF Roderick GK Olmstead KL Dobel HG (1991) Density related migration in planthoppers (Homoptera: Delphacidae): the role of habitat persistence. American Naturalist 138(6): 1513–1541. 10.1086/285298

[B10] Emeljanov AF (1996) On the question of the classification and phylogeny of the Delphacidae (Homoptera, Cicadina), with reference to larval characters. Entomological Review 75(9): 134–150. Entomologicheskoe Obozrenie 74(4): 780–794, 944–945.

[B11] Emeljanov AF (2002) Contribution to classification and phylogeny of the family Cixiidae (Hemiptera, Fulgoromorpha). Denisia 04, Zugleich Kataloge des OÖ. Landesmuseums, Neue Folge. Biologiezentrum Linz 176: 103–112.

[B12] Folmer O Black M Hoeh W Lutz R Vrijenhoek R (1994) DNA primers for amplification of mitochondrial cytochrome c oxidase subunit I from diverse metazoan invertebrates. Molecular Marine Biology and Biotechnology 3(5): 294–299. 7881515

[B13] Grevstad FS Strong DR Garcia-Rossi D Switzer RW Wecker MS (2003) Biological control of *Spartinaalterniflora* in Willapa Bay, Washington using the plant hopper *Prokelisiamarginata*: Agent specificity and early results. Biological Control 27(1): 32–42. 10.1016/S1049-9644(02)00181-0

[B14] Hernández MC Cabrera Walsh G (2014) Insect Herbivores Associated with *Ludwigia* Species, Oligospermum section, in their Argentine distribution. Journal of Insect Science 14(1): 201. 10.1093/jisesa/ieu063 25502037 PMC4684687

[B15] Kleinbaum DG Klein M (2012) Introduction to Survival Analysis. In: Survival Analysis. Statistics for Biology and Health. Springer, New York, 1–43. 10.1007/978-1-4419-6646-9_1

[B16] Mariani R Sosa A Marino de Remes Lenicov AM (2007) *Megamelusbellicus* Remes Lenicov & Sosa (Hemiptera: Delphacidae) immature stages and biology. Revista de la Sociedad Entomológica Argentina 66(3–4): 189–196. [RSEA]

[B17] Mariani R Sosa AJ Marino de Remes Lenicov AM (2013) A new species of *Megamelus* from Argentina and the reassignment of *Stenocranusmaculipes* (Berg) (Hemiptera: Delphacidae). Revista de la Sociedad Entomológica Argentina 72(3–4): 169–178. [RSEA]

[B18] Marino de Remes Lenicov AMM Defea B Rusconi J Cabrera Walsh G (2017) Studies on the immature stages of the planthopper *Lepidelphaxpistiae* (Hemiptera: Delphacidae), a potential biocontrol agent for the aquatic weed *Pistiastratiotes* (Araceae) from Argentina (Hemiptera: Fulgoromorpha). Austral Entomology 56(4): 384–391. 10.1111/aen.12248

[B19] Metcalf ZP (1923) A key to the Fulgoridae of Eastern North America with descriptions of new species. Journal of the Elisha Mitchell Scientific Society 38: 139–230. 10.5962/bhl.part.7606

[B20] Morgan LW Beamer RH (1949) A revision of three genera of Delphacine Fulgorids from America North of Mexico (Homoptera-Fulgoridac-Delphacinac). Journal of the Kansas Entomological Society 22(1): 212–241. https://www.jstor.org/stable/25081893

[B21] Muir FAG Giffard WM (1924) Studies in North American Delphacidae. Bulletin of the Experimental Station of Hawaiian Sugar Planters Association. Division of Entomology. Honolulu 15: 1–53.

[B22] Oman PW (1947) The types of Auchenorrhynchous Homoptera in the Iowa State College collection. Iowa State College Journal of Science 21: 161–228.

[B23] Paterson ID Motitsoe SN Coetzee JA Hill MP (2023) Recent post-release evaluations of weed biocontrol programmes in South Africa: A summary of what has been achieved and what can be improved. BioControl. 10.1007/s10526-023-10215-4

[B24] Peng CI (1990) Taiwan, and its origin. Botanical Bulletin of Academia Sinica 31: 343–349.

[B25] R Core Team (2022) R: A language and environment for statistical computing. R Foundation for Statistical Computing, Vienna, Austria. https://www.R-project.org/ [Accessed 1 August 2023]

[B26] Raven PH (1963) Amphitropical relationships in the floras of North and South America. The Quarterly Review of Biology 38(2): 151–177. 10.1086/403797

[B27] Reddy AM Pratt PD Grewell BJ Harms NE Cibils-Stewart X Cabrera Walsh G Faltlhauser A (2021) Biological and host range characteristics of *Lysathiaflavipes* (Coleoptera: Chrysomelidae), a candidate biological control agent of invasive *Ludwigia* spp. (Onagraceae) in the USA. Insects 12(5): 471. 10.3390/insects12050471 34069473 PMC8159108

[B28] Remes Lenicov AMM Cabrera Walsh G (2013) A new genus and species of Delphacini associated to hydrophytic plants in Argentina (Hemiptera, Fulgoromorpha, Delphacidae). The Florida Entomologist 96(4): 1350–1358. 10.1653/024.096.0414

[B29] Sosa AJ de Remes Lenicov AMM Mariani R Cordo H (2004) Redescription of *Megamelusscutellaris* Berg (Hemiptera: Delphacidae), a candidate for biological control of water hyacinth. Annals of the Entomological Society of America 97(2): 271–275. 10.1603/0013-8746(2004)097[0271:ROMSBH]2.0.CO;2

[B30] Sosa AJ de Remes Lenicov AMM Mariani R Cordo H (2005) Life history of *Megamelusscutellaris* Berg with description of immature stages (Hemiptera, Delphacidae). Annals of the Entomological Society of America 98(1): 66–72. 10.1603/0013-8746(2005)098[0066:LHOMSW]2.0.CO;2

[B31] Sosa AJ de Remes Lenicov AMM Mariani R (2007) Species of *Megamelus* (Hemiptera-Delphacidae) associated with Pontedereaceae in South America. Annals of the Entomological Society of America 100(6): 798–809. 10.1603/0013-8746(2007)100[798:SOMHDA]2.0.CO;2

[B32] Spooner CS (1912) Some new species of Delphacidae. Canadian Entomologist 44(8): 233–242. 10.4039/Ent44233-8

[B33] Stiling P Moon DC (2005) Quality or Quantity: The Direct and Indirect Effects of Host Plants on Herbivores and Their Natural Enemies. Oecologia 142(3): 413–420. 10.1007/s00442-004-1739-4 15517407

[B34] Thouvenot L Haury J Thiebaut G (2013) A success story: Water primroses, aquatic plant pests. Aquatic Conservation 23(5): 790–803. 10.1002/aqc.2387

[B35] Tipping PW Sosa A Pokorny EN Foley J Schmitz DC Lane JS Cole MS Nichols G (2014) Release and establishment of *Megamelusscutellaris* (Hemiptera: Delphacidae) on water hyacinth in Florida. The Florida Entomologist 97(2): 804–806. 10.1653/024.097.0264

[B36] Van Duzee EP (1897) A preliminary review of the North American Delphacidae. Bulletin of Buffalo Society of Natural Science 5: 225–261.

[B37] Vilbaste J (1968) Preliminary key for the identification of the nymphs of North European HomopteraCicadina. I. Delphacidae. Annales Entomologici Fennici 34: 65–74.

[B38] Wagner WL Hoch PC Raven PH (2007) Revised classification of the Onagraceae. Systematic Botany Monographs 83: 1–240.

[B39] Wilson SW Tsai JH (1991) Descriptions of Nymphs of the Delphacid Planthopper *Pissonotusdelicatus* (Homoptera: Fulgoroidea). Journal of the New York Entomological Society 99(2): 242–247. https://www.jstor.org/stable/25009897

[B40] Wilson SW Mitter C Denno RF Wilson MR (1994) Evolutionary patterns of host plant use by delphacid planthoppers and their relatives. In: Denno RF Perfect TJ (Eds) Planthoppers: their ecology and management.Springer US, Boston, 7–113. 10.1007/978-1-4615-2395-6_2

[B41] Yang CT Fang SJ (1993) Phylogeny of Fulgoromorpha nymphs, first results. In: Drosopoulos S Petrakis PV Claridge MF de Virijer PWF (Eds) Proceeding 8thAuchenorrhyncha Congress; 9–13 Aug., Delphi, Greece, 25–26.

[B42] Yang CT Yeh WB (1994) Nymphs of Fulgoroidea (Homoptera: Auchenorrhyncha) with descriptions of two new species and notes on adults of Dictyopharidae. Chinese Journal of Entomology, Special Publication 8, 189 pp.

[B43] Zardini EM Gu H Raven PH (1991) On the separation of two species within the *Ludwigiauruguayensis* complex (Onagraceae). Systematic Botany 16(2): 242–244. 10.2307/2419276

