# Supplementary material for: ﻿New morphological and biological contributions to adults and immature forms of Pissonotusparaguayensis (Fulgoromorpha, Delphacidae) in wetlands of Argentina
Source: Zookeys. 2024 Jan 8;1188:227–50. doi: 10.3897/zookeys.1188.113350 (PMC10790368; doi:10.3897/zookeys.1188.113350)
Supplement: Supplementary material 1 — Graphical figures of Kaplan-Meier curves of Pissonotusparaguayensis males and female survival probability on different test plants [file zookeys-1188-227_article-113350__-s001.docx]

**SUPPLEMENTARY MATERIAL 1**

**New morphological and biological contributions to adults and immature forms of *Pissonotus paraguayensis* (Fulgoromorpha, Delphacidae) in wetlands of Argentina**

Marino de Remes Lenicov, Ana M ^1,2^, Faltlhauser, Ana C^2,3,4^, Foieri, Alvaro^1,5^, Salinas, Nicolas A^2,3,4^, Hernández, M. Cristina^3^ & Sosa, Alejandro J ^2,3^

**
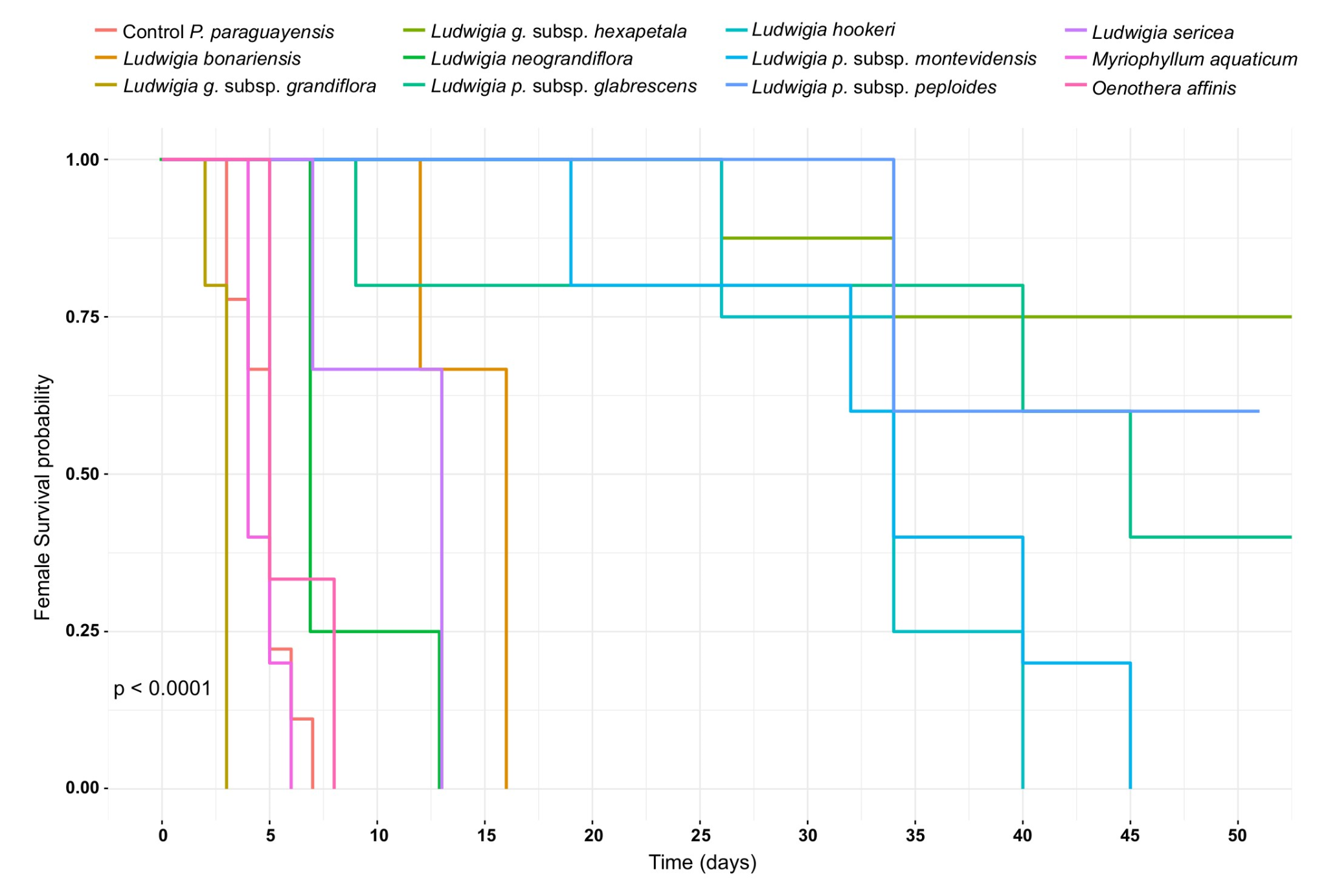
Figure S1:** Kaplan-Meier curves of female *Pissonotus paraguayensis* survival probability on different test plants (colours). Non-parametric Log-rank statistical test was used to assess statistical differences in overall survival.

**
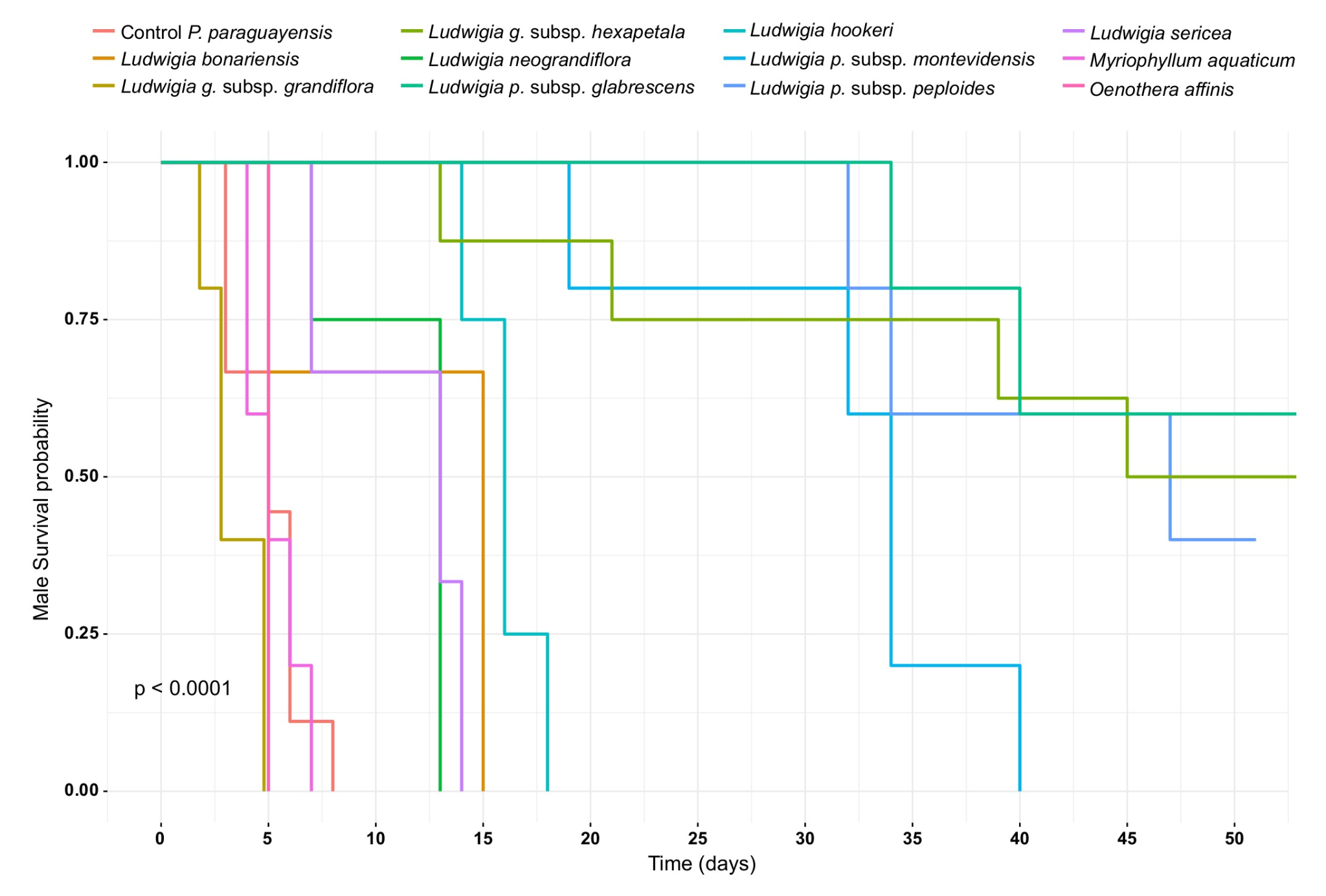
**

**Figure S2:** Kaplan-Meier curves of male *Pissonotus paraguayensis* survival probability on different test plants (colours). Non-parametric Log-rank statistical test was used to assess statistical differences in overall survival.
